# Impacts of Dietary Protein and Prebiotic Inclusion on Liver and Spleen Gene Expression in Hy-Line Brown Caged Layers

**DOI:** 10.3390/ani10030453

**Published:** 2020-03-09

**Authors:** Morouj N. Al-Ajeeli, Shawna M. Hubert, Hector Leyva-Jimenez, Mohammed M. Hashim, Raghad A. Abdaljaleel, Akhil M. Alsadwi, Giridhar Athrey, Christopher A. Bailey

**Affiliations:** 1 Department of Poultry Science, Texas A&M University, College Station, TX 77843-2472, USA; morouj@aggienetwork.com (M.N.A.-A.); smhubert@mdanderson.org (S.M.H.); texas53297@tamu.edu (H.L.-J.); hashim@aggienetwork.com (M.M.H.); raghad20042002@yahoo.com (R.A.A.); akhil_vet@yahoo.com (A.M.A.); c-bailey@tamu.edu (C.A.B.); 2 Calpis America, Inc. 455 Dividend Dr, Peachtree, GA 30269, USA

**Keywords:** laying hens, yeast cell wall, soybean meal, cottonseed meal, prebiotic

## Abstract

**Simple Summary:**

Eggs are one of the most affordable and nutritious animal proteins available, and with increasing human population, there is an increased demand for production. As feed is the main expense in poultry production, novel protein sources and feed additives need to be evaluated for their benefits for poultry health and performance. In this study, we evaluated the standard soybean-based diets against an alternate source—cottonseed meal, in the context of prebiotic addition. Prebiotics putatively improves health and production. We assessed the homeostatic and immune balance by assaying the expression of select marker genes. We find that the inclusion of yeast cell wall products as prebiotic alters homeostatic balance. Particularly, the upregulation of apoptosis—a normal cell process—suggests that these products may promote homeostatic balance.

**Abstract:**

The ingredients of poultry feeds are chosen based on the least-cost formulation to meet nutritional requirements. However, this approach can lead to the introduction of anti-nutritional ingredients in the feed. The objective of this study was to evaluate the impacts of two diets (with or without prebiotic) on homeostatic genes in the liver and spleen of laying hens. Hy-Line Brown layers were raised either on a soybean meal or cottonseed meal-based diets with and without an added prebiotic (yeast cell wall), totaling four experimental diets. A total of 120, 63-week old layers were housed individually in a wire cage system. We investigated differences in the expression of select homeostatic marker genes in the liver and spleen of hens from each treatment. We then used the ΔΔCT and generalized linear models to assess significance. Results show that the inclusion of prebiotic yeast cell-wall (YCW) increased the expression of the *BAK* gene in the liver tissue for both the soybean meal (SBM) and cottonseed meal (CSM) diets. For splenic tissue, the combination of YCW with the CSM diet increased the POR gene over six log2 fold. Altogether, our results suggest altered homeostasis, which can have consequences for health and performance.

## 1. Introduction

Chicken meat and eggs are an important and economical source of animal protein around the world. Furthermore, the production of chicken meat and eggs is efficient and has a smaller ecological footprint than beef and pork production [1]. The efficiency of chicken production is in part due to its dietary flexibility, allowing the industry to utilize feed components based on the least-cost formulation [2]. The majority of industry broiler and layer diets are primarily composed of corn and soybean meal (protein source), together with synthetic vitamins, minerals, and supplemental enzymes to improve digestibility. Soybean meal (SBM) is produced during the processing of soybean, where the protein concentrate, oil, and other components are extracted. It is considered a great source of protein [3] as well as a means of balancing dietary amino acid levels with other ingredients [4]. However, the cost of SBM is volatile and can increase feed costs dramatically [5]. Raw soybeans also have several anti-nutrient factors such as trypsin inhibitors, lectins, saponins, and stachyose and raffinose non-starch polysaccharides. Therefore, there is a need to evaluate the benefits of alternative protein sources for livestock feed. One energy-rich alternative to SBM is cottonseed meal [6].

Cottonseed meal (CSM) contains approximately 41% protein, 13.6% crude fiber, and 0.5% crude fat (NRC, 1994). It is derived from the residue created during cottonseed oil extraction [7], similar to SBM production. CSM is usually less expensive than SBM, but the former also contains antinutritional compounds that potentially limit its use. Furthermore, concerns regarding gossypol toxicity and cyclopropenoid fatty acids limit the use of this product in all poultry diets [8]. The main anti-nutritional factor of CSM is free gossypol, which lowers protein digestibility and can affect the reproductive system, heart, and liver in monogastric animals [7] leading to decreased poultry performance [9].

Generally, laying hens are more susceptible to free gossypol than other poultry, as there are several negative impacts on egg quality parameters from ingestion of free gossypol and cyclopropenoid fatty acids [10], and an overall reduction in performance [9]. Free gossypol can also cause discoloration in the yolk due to the chemical combination between ferric ions that are released from yolk protein and the gossypol compound [11]. Dietary levels of free gossypol up to 50 parts per million (ppm) are considered safe, without egg yolk discoloration [12].

One approach to mitigate these properties is with prebiotic usage. Prebiotics are defined as non-digestible ingredients that selectively stimulate the presence of beneficial bacteria in the colon, resulting in improvement of the host’s health [13]), improved live weight, and reduced feed conversion ratio [14]). As a result, there is a growing interest in the use of prebiotics to improve general gut health and feed utilization [15]. However, the consequences of prebiotic administration on homeostasis remains unclear.

Mannan oligosaccharides are classified as prebiotic due to their reported anti-pathogenic properties that interfere with the colonization of bacteria with mannose-seeking lectins [16]). Mannan oligosaccharides, mannoproteins, and β-glucans are components of the outer layer of yeast cell walls (YCW) of *Saccharomyces cerevisiae* and are increasingly used as a prebiotic feed additive to elicit benefits for the gut health and improved productivity. YCW products are now widely used as prebiotics in feed for layers, broilers, and other poultry. For instance, supplementation of YCW-mannan oligosaccharide in layer diets has been found to significantly improve the feed and the caloric conversion ratio [17].

The objective of this study was to evaluate markers of homeostatic balance in layer chicken raised on SBM versus CSM meals and supplemented with YCW as a prebiotic. Our study was prompted by recent interest in alternative protein sources in animal feed, and the inclusion of prebiotic additives in animal production. While such assessments are primarily focused on comparing growth and production performance, there is increasing interest in determining the physiological and immune parameters. Several fish studies have focused on this [18,19,20]), but to our knowledge, data on chicken homeostatic gene expression are not available. This study addresses this gap. The overarching hypothesis was that both the dietary protein source and prebiotic addition induce notable homeostatic and immune gene changes, as assayed in the liver and spleen tissues, respectively.

This study is part of a group of studies that assessed the suitability of SBM and CSM based feeds for poultry production. In previously published studies, comparisons of live production performance and gut-microbiota have been presented [21,22]). In this study, we investigated how prebiotic administration alters systemic processes—as measured by gene expression, in the context of SBM versus CSM based feeds. Specifically, we characterized gene expression profiles of select genes in the liver and spleen in caged laying hens raised on four diet combinations. It is important to note that commercial feed for livestock production contains hundreds of ingredients, and it is not possible to truly pinpoint the causative factors for differences in gene expression. Nonetheless, our experimental design (2 × 2 factorial) was designed to help narrow down the source of the differences arising from the basal diets (SBM vs. CSM) from the interactive effects generated by prebiotic inclusion (YCW vs. no additive). The findings of the study are informative about the implications of dietary protein and prebiotic inclusion for health and homeostasis in laying hens. Taken together with previous reports, this study can provide valuable information about the suitability of alternative feed ingredients and prebiotic additives in livestock production.

## 2. Materials and Methods 

### 2.1. Live Animal Study

This study was performed at the Texas A&M University Poultry Research Center, following protocols approved by the University’s Animal Care and Use Committee (IACUC 2017-0072). A total of 120 layers at 63 weeks were used in this study. These older hens were used for this study as the birds were part of the performance study reported in [21], and could not be euthanized at an earlier point. Hens were distributed in wire cages a 50.8 cm W × 30.5 cm L × 30.5 cm H per cage (1549 cm^2^/hen) with one nipple drinker for every two cages in a randomized block design. Each cage had access to individual trough feeders (30.5 cm feeder space/hen), with ad libitum feed and water. The diets were formulated based on the nutrient requirements suggested by the 2014 Hy-line Brown management guide. YCW supplemented as Safmannan^®^ was sourced from Phileo-Lesaffre Animal Care (Milwaukee, WI). Safmannan YCW was different from most other commercial products in that the YCW had a guaranteed minimum content of 20% mannan and 20% beta-glucan. Table 1 contains the full nutrient composition for each diet used in the study. All diets were made at the Texas A&M University Poultry Center Feed Mill. The hens were placed in the same dietary treatment groups from the beginning of the production period. The YCW supplementation was included from 47 weeks to 62 weeks of age (end of the study).

Laying hens were raised on a total of 4 diets: SBM, SBM + YCW, CSM, and CSM + YCW. As shown by the abbreviations above, the YCW treatment (25 mg/kg) was overlaid with the SBM, and CSM dietary treatments to create a 2 × 2 factorial arrangement of 3 randomized complete pen location blocks throughout the hen house (10 hens per treatment block). In the treatments with the prebiotic, YCW was added to the feed before pelleting. We tested the hypothesis that the expression of genes associated with homeostatic balance was not different among treatment groups, as measured by differential gene expression analyses.

### 2.2. Tissue Collection and Storage

A total of 16 Hy-line Brown laying hens (63 weeks of age; 4 per treatment) were euthanized using CO_2_ gas, and from each hen, approximately two grams of both liver and spleen tissues were collected and stored in RNALater following the manufacturer guidelines (ThermoFisher Scientific, Waltham, MA, USA). These samples were then stored at 4 °C for 24 h before being removed from the RNALater and stored at −80 °C until RNA isolation.

### 2.3. Genes for Targeted Differential Expression

To evaluate the effects of xenobiotic compounds on homeostatic balance, we selected a total of 10 genes of interest. The selected genes were associated with known pathways related to xenobiotic metabolism or as established biomarkers of homeostatic balance and apoptotic activities [23,24,25,26,27]. As the reference genes, 2 genes were used, *AHR* and *GAPDH*, which were both commonly used housekeeping genes. Table 2 contains the relevant information about the genes selected for this analysis.

### 2.4. RNA Extraction, Quality Analysis, and Reverse Transcription

Total RNA was isolated from 100 mg sections of liver and spleen tissue samples using the TRIzol Reagent method (ThermoFisher Scientific, Waltham, MA, USA), and samples were quantified on a Nanodrop Spectrophotometer (ThermoFisher, Waltham, MA, USA). The quality of the RNA isolates was checked using the Agilent Bioanalyzer 2100 (Agilent, Santa Clara, CA, USA) with the RNA 6000 Nano Kit following the manufacturer’s protocol. Samples with an RNA Integrity Number (RIN) above 7 were retained for further analyses. RNA isolates not meeting these quality criteria were re-extracted. Finally, reverse transcription reactions were performed according to the manufacturer’s protocol with SuperScript VILO Master Mix (ThermoFisher Scientific, Waltham, MA, USA). Pooled cDNA samples were used as templates for primer testing.

### 2.5. Primer Design and Testing

Primers were designed using the NCBI Primer-BLAST online tool (Table 3). Amplicon size was set to 200–300 base pairs and primers were selected to span exon-exon junctions, to exclude the probability of DNA contamination in RNA isolates. Primers were obtained from Integrated DNA Technologies (IDT, Coralville, IA, USA). We performed primer testing on a pooled cDNA sample according to the manufacturer specified protocol for the PowerUP SYBR Green Master Mix (ThermoFisher Scientific, Waltham, MA, USA). Primer pairs yielding efficiency between 90% and 110% were accepted, and we visually inspected all dissociation curves for evidence of amplification of unintended targets.

### 2.6. Real-Time Quantitative Polymerase Chain Reaction (RT-qPCR)

The RT-qPCR reactions were performed using the PowerUP SYBR Green Master Mix manufacturer’s protocol (ThermoFisher Scientific, Waltham, MA, USA). We generated expression data for each gene by reactions run concurrently on a single 384 well plate on an ABI 7900 HT (Applied Biosystems, Foster City, CA, USA). We used data from 2 duplicate reactions for further analysis.

### 2.7. Statistical Analysis

We calculated the log2 fold change (L2FC) of expression for each gene using the ΔΔCT method [28,29] in Microsoft Excel. We also tested for statistical significance of gene expression among experimental groups using the ‘aov’ procedure on the R statistical platform [30], with the treatments as the independent variable and the ΔCT values as the dependent variable (two-way ANOVA). The dietary protein source (SBM or CSM) and the prebiotic inclusion (YCW or no-additive) were the main model terms, in addition to the interaction between them. We performed post-hoc pairwise comparisons of all possible combinations using the TukeyHSD function in R. Statistical comparisons that yield a *p*-value smaller than α = 0.05 were considered significantly different.

## 3. Results

**Figure 1:**
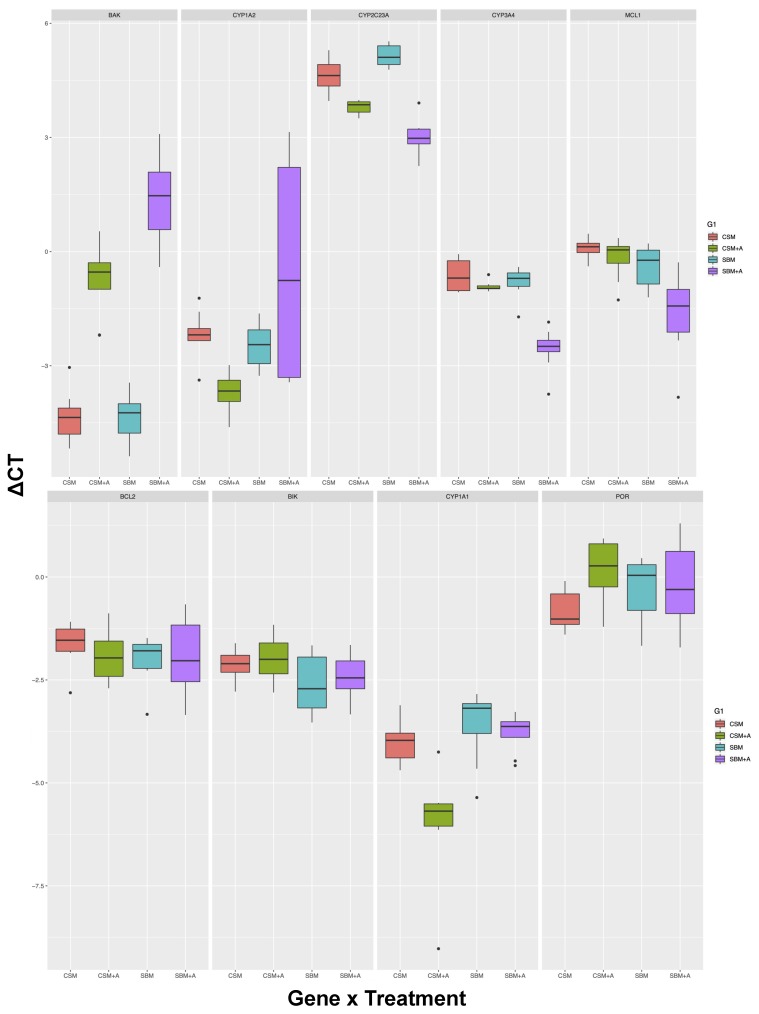
Boxplots showing the differential expression of homeostatic genes in the livers of laying hens raised on SBM, CSM, SBM + YCW, and CSM + YCW diets. The y-axis values are ΔCTs, and each dietary treatment is represented by different colors. The gene of interest is named at the top of each panel.

**Figure 2:**
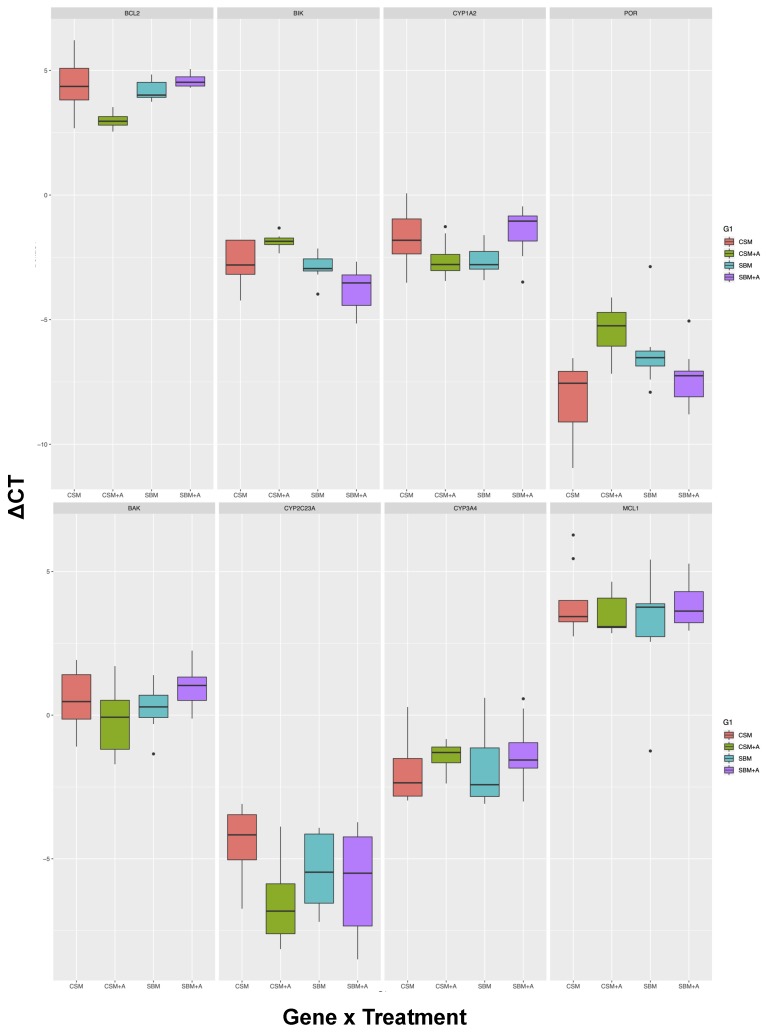
Boxplots showing the differential expression of homeostatic genes in the spleens of laying hens raised on SBM, CSM, SBM + YCW, and CSM + YCW diets. The y-axis values are ΔCTs, and each dietary treatment is represented by different colors. The gene of interest is named at the top of each panel.

Of the 10 genes assayed for this investigation, 9 yielded high-quality data, where both biological and technical replicates were represented. NLRP3 was excluded from further analyses as this locus did not pass primer testing because of weak amplification in most samples and between duplicate reactions, resulting in the inability to calculate its efficiency. Therefore, the following results and discussion focus on these 9 genes. Regardless of diet type (SBM or CSM), the addition of YCW resulted in the up-regulation of genes that are responsible for apoptosis regulation and mitochondrial energy homeostasis in liver and spleen tissues. On the other hand, genes involved in xenobiotic metabolism were both up and down-regulated (Figure 1 and Figure 2).

The 2-Way ANOVA showed an interesting pattern of differences in the liver tissue in relation to the protein source, the prebiotic supplement, and their interactions. First of all, there was a significant difference between the SBM and the CSM diets, as well as between the YCW and no-additive diets (main models). The BAK gene was significantly upregulated in both SBM + YCW and CSM + YCW diets, relative to the diets without additives (*p* < 0.01). Furthermore, the SBM + YCW diet was also significantly different from CSM + YCW. However, the expression of this gene was not different between SBM and CSM diets with no additive, suggesting an interaction between the protein source and the prebiotic (Table 4). The gene *CYP2C23A* was another gene with broad-ranging differences. While the main model term for dietary protein was not significant, the inclusion of additive, and interactive effects (post-hoc tests) were all significant. At the other extreme, the genes *BCL2* and *BIK* were least affected by variations in dietary source or prebiotic additives. Across all tested genes, the major pattern, when differences were found, was that there were differences between the dietary protein source in the presence of YCW additive (Table 4).

In the spleen, highly different outcomes were seen compared to the liver. *BCL2* and *BIK* were the two genes that generated the greatest responses. In both these cases, the overall dietary protein source (main model) was significant, but the YCW addition was only significant for *BCL2*. Furthermore, for both these genes, some of the post-hoc pairwise comparisons returned significant differences; importantly, they showed that the YCW interactive effect was absent, in contrast to the results seen in the liver (Table 4). Overall, spleen tissue was affected to a much lower degree than the liver tissue. In summary, the major findings of our analysis were that the liver shows significant responses to the dietary protein source, the addition of prebiotic YCW, as well as an interactive effect between the dietary protein and prebiotic. In comparison, the spleen was relatively unresponsive to these dietary additives.

## 4. Discussion

There is increasing interest in the inclusion of YCW as prebiotic in animal feed due to its property as a functional feed [18]. The presence of various potentially beneficial components in YCW makes it potentially useful for improving performance and health. In aquaculture, for example, their role in reducing sea-louse parasitism to improving immunity has been investigated [18,20] In dogs, the inclusion of YCW helped improve gut health and generated positive immunomodulatory effects [31]. A study on broilers showed improved intestinal immunoglobulin A and immune responses in feeds with YCW inclusion [32]. These studies emphasize the significance and relevance of the assessments presented in this study. Additionally, they justify the need to investigate the effects of YCW in different feed combinations and livestock models (laying hens here).

Our investigation demonstrated that the YCW prebiotic supplement alters gene expression of multiple xenobiotic metabolism genes and apoptosis-regulating genes. While we found that homeostatic gene expression in the liver was highly responsive to dietary protein and YCW inclusion, gene expression in the spleen was relatively less responsive. Specifically, the up-regulation of *BAK* in the liver appears to be driven by the YCW prebiotic, as this interaction was absent in the comparison between the SBM and CSM diets without YCW. It is of interest that the *BAK* gene is responsible for apoptosis regulation through mitochondrial membrane pore formation [33,34,35].

BAK is a member of the *BCL2* gene family. The *BCL2* gene family consists of both pro- and anti-apoptotic members [24,33,35]. Other members of this family, which we investigated here, include *MCL1*, *BIK*, and *BCL2*. The *MCL1* and *BCL2* are anti-apoptotic while *BIK* is pro-apoptotic [24,36,37]. The *BIK* gene targets the endoplasmic reticulum (ER) membrane [38] It is an accelerator of programmed cell death, and this occurs via apoptosis. Apoptosis plays a crucial role in developmental regulation and tissue homeostasis [24]. Viedma-Rodriguez et al. [34] showed BIK as a reliable therapeutic molecule in gene therapy-based approaches to treat cancer, and researchers have found that this suppression of the gene enhances resistance to tamoxifen in *MCF-7* breast cancer cells. Our study shows that the YCW prebiotic not only increases *BAK* expression but also decreases MCL1 expression in the liver, indicating increased levels of apoptosis by Cytochrome-c release from the mitochondria. This outcome is similar between the SBM + YCW and CSM + YCW diets, although the impact of YCW is more pronounced in the SBM + YCW diet. In salmonid fish, the addition of YCW also resulted in increased immune gene expression related to chemokine signaling in the liver [20].

The gene expression observed in the spleen is more dependent on the protein source used in the diet. Overall, the *BCL2* and *BIK* genes were the most active in the spleen. Significantly elevated *BLC2* (anti-apoptotic) expression was observed in the SBM + YCW diet, whereas *BIK* (pro-apoptotic) expression was significantly decreased. The opposite pattern was observed for the CSM + YCW treatment group. The opposing directional expression of these genes (*BCL2* and *BIK*, Figure 2) in the spleen indicates that the CSM + YCW diet is eliciting pro-apoptotic signaling while the SBM + YCW diet is eliciting anti-apoptotic signaling.

Multiple members of the Cytochrome P450 family were also investigated here and showed altered expression due to the YCW supplement. These genes were included due to their activities in xenobiotic metabolism [26,27]. Many xenobiotics can reach toxic concentrations without adequate metabolism [39]. The Cytochrome P450 (CYP) families 1-3 are recognized as the critical xenobiotic-metabolizing enzymes that participate in the bioactivation, or the inactivation of different xenobiotics compounds [26,27]. The *CYP1A2* gene plays a significant role in the metabolism of several drugs as well as carcinogen activation because of its higher level of expression in the liver [26]. These genes also play an essential role with NADPH- dependent electron transport pathway as *CYP1A1* is considered a monooxygenase for xenobiotic and drug metabolism, and *CYP1A2* participates in the bioactivation of carcinogens.

The POR gene is also a member of the P450 family. It acts as an oxidoreductase containing flavin adenine dinucleotide and flavin mononucleotide moieties that transfer electrons from NADPH to microsomal cytochrome P450 enzymes [40]. It is generally involved in the oxidative metabolism of steroids and carcinogens. A deficiency of POR can cause disordered steroidogenesis, which is associated with mutations causing genital ambiguity in both sexes [41] Researchers have examined the biological function of Cytochrome P450 in mouse small intestine and confirmed the relation or the mechanistic link between intestinal immunity and the POR-dependent enzymes [42].

Interestingly, while the genes selected for this investigation were picked to determine the homeostatic effects of anti-nutrient components in the SBM or CSM diets, they revealed significant effects of the YCW supplement as well. Specifically, in the liver, the inclusion of YCW significantly downregulated all members of the Cytochrome P450 family investigated except for POR. These findings show that xenobiotic metabolism may be decreased under these conditions. The up-regulation of POR in the spleen was also observed due to YCW supplementation. Our results indicated that the use of a diet utilizing CSM caused significant changes in the expression of several genes in the liver, and to a lesser extent in the spleen. Based on the Insect Control and Cotton Disease Research Unit/Southern Plains ARS/USDA gossypol analysis of the CSM diet, the free gossypol level was below 21.8 ppm, which is not typically considered a toxic concentration. Therefore, our results indicate a role for other compounds in CSM or the interactive effect of gossypol in these diets. However, this study did not investigate these effects specifically.

## 5. Conclusions

In this study, we found that regardless of the diet type (SBM or CSM), the prebiotic YCW supplementation elicits the expression of BAK in the liver tissue, indicating a pro-apoptotic environment. As apoptosis is a normal, homeostatic process, the results indicate that the addition of YCW supports homeostatic balance. Furthermore, our results demonstrate an interactive effect of YCW with both the SBM and CSM based diets. Therefore, the use of YCW in poultry feeds needs to be further investigated regarding other measurements of production and welfare due to increases in pro-apoptotic and xenobiotic gene expression.

## Figures and Tables

**Table 1:** Composition and nutrient levels of soybean meal (SBM) and cottonseed meal (CSM) diets with or without 250 ppm yeast cell-wall (YCW) for Hy-Line Brown Layers (47 to 62 weeks of age).

Ingredients	47–55 Weeks of Age		55–62 Weeks of Age	
	SBM%	CSM%	SBM%	CSM%
Corn	64.17	42.22	65.67	51.19
Dehulled Soybean Meal	21.17	0	19.56	0
Cottonseed Meal	0	15	0	15
Corn Gluten Meal	0.38	1.66	0.29	1.14
Dried Distiller Grains	0	15	0	15
Wheat Middlings	0	8.31	0	1.08
DL-Methionine 98%	0.17	0.22	0.15	0.16
L-Threonine 98%	0	0.06	0	0
Lysine HCL	0	0.48	0.02	0.43
AV ^4^ Fat Blend	2.12	5	1.27	2.74
Limestone	9.91	10.1	11	11.1
Dicalcium Phosphate	1.42	1.37	1.43	1.44
Salt	0.38	0.04	0.33	0.04
Sodium Bicarbonate	0	0.27	0.03	0.37
Trace Minerals ^1^	0.05	0.05	0.05	0.05
Vitamins ^2^	0.25	0.25	0.25	0.25
Calculated Nutrient Composition (%)				
ME (kcal/kg)	2867	2867	2800	2800
Crude Protein	16.75	16.75	16	16
Crude Fat	3.95	7.66	3.13	5.4
Crude Fiber	1.75	4.47	1.71	4.05
Calcium	4.1	4.1	4.5	4.5
Phosphorous	0.64	0.75	0.63	0.73
AV Phosphate	0.4	0.4	0.4	0.4
AV Methionine ^3^	0.41	0.44	0.38	0.38
AV Lysine ^3^	0.74	0.77	0.71	0.71
AV TSAA ^3^	0.64	0.67	0.6	0.6

SBM% = Soybean meal diet ingredients percentages; CSM% = Cottonseed meal diet ingredients percentages. ^1^ Trace minerals premix added at this rate yields (mg/kg): Zinc, 60.0; manganese, 60.0; iron, 60.0; copper, 7.0; iodine, 0.4. ^2^ Vitamin premix added at this rate yields (per kg): Vitamin A, 11 IU; vitamin D3, 3,850 IU; vitamin E, 45.8 IU; menadione, 1.5 mg; B12, 0.017 mg; biotin, 0.55 mg; thiamine, 2.93 mg; riboflavin, 5.96 mg; d-pantothenic acid, 20.17 mg; B6, 7.15 mg; niacin,45.8 mg; folic acid, 1.74 mg; choline, 130.3 mg. ^3^ Standardized digestibility Coefficients for cottonseed Methionine, Lysine, and TSAA (Total Sulfur Amino Acids) were 0.73, 0.67, and 0.73, respectively. AV refers to available. ^4^ AV Fat Blend: Animal-Vegetable Blended Fat.

**Table 2:** List of selected genes. Reference genes are denoted by (R) next to the gene abbreviation.

Gene Name	Abbreviation	Function
*Aryl Hydrocarbon Receptor*	*AHR (R)*	Cell-cycle regulation and tissue development
*Glyceraldehyde-3-phosphate Dehydrogenase*	*GAPDH (R)*	Glycolysis, RNA transport, and DNA replication
*BCL2 Agonist/Killer*	*BAK*	Apoptosis regulation, mitochondria energy metabolism regulation
*BCL2 Interacting Killer*	*BIK*	Programmed cell death accelerator via apoptosis
*B-Cell Lymphoma 2 Apoptosis Regulator*	*BCL2*	Apoptosis suppressor, cell death regulator
*Myeloid Leukemia Apoptosis Regulator*	*MCL1*	Anti-apoptotic protein, cell viability maintenance
*Nucleotide-Binding Leucine-Rich Repeat Protein*	*NLRP3*	Innate immunity
*P450 Oxidoreductase*	*POR*	Oxidative metabolism of steroids, and carcinogens
*Cytochrome P450 Family 1 Subfamily A Member 1*	*CYP1A1*	NADPH-dependent electron transport pathway
*Cytochrome P450 Family 1 Subfamily A Member 2*	*CYP1A2*	Xenobiotic metabolism, carcinogenic aromatic
*Cytochrome P450 2C23A*	*CYP2C23A*	Xenobiotic and drug metabolism
*Cytochrome P450 3A4*	*CYP3A4*	Monooxygenase

**Table 3:** Forward and reverse primer sequences for each gene used for real-time quantitative Polymerase Chain Reaction (RT-qPCR).

Gene	Primer Sequence 5′–3′	
*BAK*	Forward Primer	ACGAGAGATCAATGCAGAGGAC
	Reverse Primer	ACTCGTAGGCGTTCTCCTTG
*BIK*	Forward Primer	TCTCCAGATACCCCAACGGA
	Reverse Primer	ACTGATAGCAACCCTGCGTG
*BCL2*	Forward Primer	GGATGGGATGCCTTTGTGGAA
	Reverse Primer	TTAGCCAGGAAGTTGTTTTGCTC
*MCL1*	Forward Primer	GAGGCTGGGAGGGCTTTGTT
	Reverse Primer	GGTGACTCAAGTCTGGCTGT
*NLRP3*	Forward Primer	GTCACTAAACCTGGTGGGGC
	Reverse Primer	CCTGCGCTCTCCTGATCCAT
*POR*	Forward Primer	ACAAGGGAAGTGAGTGGAGTT
	Reverse Primer	ACTATGTTTCGGCCCGTCTT
*CYP1A1*	Forward Primer	GCAGCACCCAAAGGTTCACT
	Reverse Primer	ATGGTCACCTCCATCACGTC
*CYP1A2*	Forward Primer	ACACCACGCTTCCCCTTAGT
	Reverse Primer	TCCATCACGTCCCCGTATTT
*CYP2C23A*	Forward Primer	CCTTCAGTGGGAGAGGAATACTG
	Reverse Primer	TGAAAGGTTCCTCGTGTGTCTT
*CYP3A4*	Forward Primer	ACACCACGCTTCCCCTTAGT
	Reverse Primer	TCCATCACGTCCCCGTATTT
*AHR*	Forward Primer	GTGCAGAAAATAGTAAAGCCATCT
	Reverse Primer	CCCTCTCCAAGTTTTGCTGT
*GAPDH*	Forward Primer	TCGGAGTCAACGGATTTGGC
	Reverse Primer	GCCCATTTGATGTTGCTGGG

**Table 4:** Summary of 2-Way ANOVA results based on two main variables—dietary protein (SBM vs. CSM) and prebiotic inclusion (YCW vs. no additive), and the interaction term. In addition, presented are the summary of post-hoc pairwise analyses using the Tukey Honest Significance (HSD). The count of significant pairwise comparisons are given in column six, and the non-significant results of pairwise comparisons are listed in the Post-hoc N.S column.

Tissue	Gene	SBM vs. CSM	YCW vs. No Add	Interaction	Significant Post-hoc Tests	Post-hoc N.S
Liver	*BAK*	<0.01	<0.001	<0.01	5 out of 6	SBM v.s CSM
	*CYP1A2*	<0.05	n.s	<0.01	1 out of 6	SBM vs CSM, CSM + YCW vs. CSM, SBM + YCW vs. SBM, CSM + YCW vs. SBM, SBM vs. SBM + YCW
	*CYP2C23A*	n.s	<0.001	<0.001	6 out of 6	All pairs different
	*CYP3A4*	<0.001	<0.001	<0.001	3 out of 6	SBM vs. CSM, CSM vs. CSM + YCW, CSM + YCW vs. SBM
	*MCL1*	<0.001	<0.01	n.s	3 out of 6	SBM vs. CSM, CSM vs. CSM + YCW, CSM + YCW vs. SBM
	*BCL2*	n.s	n.s	n.s	0 out of 6	All pairs not different
	*BIK*	<0.05	n.s	n.s	0 out of 6	All pairs not different
	*CYP1A1*	<0.01	<0.01	<0.05	3 out of 6	SBM vs. CSM, SBM + YCW vs. CSM, SBM + YCW vs. SBM
	*POR*	n.s	n.s	n.s	0 out of 6	All pairs not different
**Tissue**	**Gene**	**SBM vs. CSM**	**YCW vs. No Add**	**Interaction**	**Significant Post-hoc Tests**	**Post-hoc N.S**
Spleen	*BAK*	n.s	n.s	n.s	0 out of 6	All pairs not different
	*CYP1A2*	n.s	n.s	<0.01	0 out of 6	All pairs not different
	*CYP2C23A*	n.s	<0.05	n.s	0 out of 6	All pairs not different
	*CYP3A4*	n.s	n.s	n.s	0 out of 6	All pairs not different
	*MCL1*	n.s	n.s	n.s	0 out of 6	All pairs not different
	*BCL2*	<0.01	<0.05	<0.001	3 out of 6	SBM vs CSM, SMB + YCW vs. CSM, SBM + YCW vs. SBM
	*BIK*	<0.001	n.s	<0.01	3 out of 6	SBM vs. CSM, CSM + YCW vs. CSM, SMB + YCW vs. SBM
	*CYP1A1*	ND	ND	ND	ND	ND
	*POR*	n.s	n.s	<0.001	2 out of 6	SBM vs. CSM, SMB + YCW vs. CSM, CSM + YCW vs. SBM, SBM + YCW vs. SBM
